# Follow-Up Genotoxic Study: Chromosome Damage Two and Six Years after Exposure to the Prestige Oil Spill

**DOI:** 10.1371/journal.pone.0132413

**Published:** 2015-07-29

**Authors:** Kristin Hildur, Cristina Templado, Jan-Paul Zock, Jesús Giraldo, Francisco Pozo-Rodríguez, Alexandra Frances, Gemma Monyarch, Gema Rodríguez-Trigo, Emma Rodriguez-Rodriguez, Ana Souto, Federico P. Gómez, Josep M. Antó, Joan Albert Barberà, Carme Fuster

**Affiliations:** 1 Unitat de Biologia Cel·lular i Genètica Mèdica, Facultat de Medicina, Universitat Autònoma de Barcelona (UAB), Bellaterra, Spain; 2 Centre de Recerca en Epidemiologia Ambiental (CREAL), Barcelona, Spain; 3 Hospital del Mar Medical Research Institute, Barcelona, Spain; 4 CIBER Epidemiologia i Salut Pública (CIBERESP), Barcelona, Spain; 5 Unitat de Bioestadística and Institut de Neurociències, Facultat de Medicina, UAB, Bellaterra, Spain; 6 Departamento de Medicina Respiratoria, Unidad Epidemiologia Clínica, Hospital 12 de Octubre, Madrid, Spain; 7 CIBER Enfermedades Respiratorias (CIBERES), Bunyola, Mallorca, Spain; 8 Departamento de Medicina Respiratoria, Hospital Clínico San Carlos, Madrid, Spain; 9 Departamento de Medicina Respiratoria, Complexo Hospitalario Universitario A Coruña, A Coruña, Spain; 10 Departament de Medicina Respiratòria, Hospital Clínic-Institut d’Investigacions Biomèdiques August Pi i Sunyer (IDIBAPS), Barcelona, Spain; 11 Universitat Pompeu Fabra (UPF), Barcelona, Spain; University at Buffalo, UNITED STATES

## Abstract

**Background:**

The north-west coast of Spain was heavily contaminated by the *Prestige* oil spill, in 2002. Individuals who participated in the clean-up tasks showed increased chromosome damage two years after exposure. Long-term clinical implications of chromosome damage are still unknown.

**Objective:**

To realize a follow-up genotoxic study to detect whether the chromosome damage persisted six years after exposure to the oil.

**Design:**

Follow-up study.

**Setting:**

Fishermen cooperatives in coastal villages.

**Participants:**

Local fishermen who were highly exposed (n = 52) and non-exposed (n = 23) to oil seven years after the spill.

**Measurements:**

Chromosome damage in circulating lymphocytes.

**Results:**

Chromosome damage in exposed individuals persists six years after oil exposure, with a similar incidence than those previously detected four years before. A surprising increase in chromosome damage in non-exposed individual was found six years after *Prestige* spill vs. those detected two years after the exposure.

**Limitations:**

The sample size and the possibility of some kind of selection bias should be considered. Genotoxic results cannot be extrapolated to the approximately 300,000 individuals who participated occasionally in clean-up tasks.

**Conclusion:**

The persistence of chromosome damage detected in exposed individuals six years after oil exposure seems to indicate that the cells of the bone marrow are affected. A surprising increase in chromosome damage in non-exposed individuals detected in the follow-up study suggests an indirect exposition of these individuals to some oil compounds or to other toxic agents during the last four years. More long-term studies are needed to confirm the presence of chromosome damage in exposed and non-exposed fishermen due to the association between increased chromosomal damage and increased risk of cancer. Understanding and detecting chromosome damage is important for detecting cancer in its early stages. The present work is the first follow-up cytogenetic study carried out in lymphocytes to determine genotoxic damage evolution between two and six years after oil exposure in same individuals.

## Introduction

In 2002, the oil tanker *Prestige* wrecked offshore of the Spanish North-West (Galicia) and thousand tons of crude oil were spilled contaminating more than 1,000 km of coast [1]. Fishermen in local areas took an active role in the clean-up tasks without using any kind of exposure protection to the toxic components of oil, some of which are considered carcinogenic [2,3]. Most oil exposure studies on health effects have been focused on physiological symptoms [4,5] and relatively little is known about the existence of a relationship between exposure to oil and genotoxic damage [6–16].

The effects of oil spills on human health have mainly focused on the acute phase. Most studies described respiratory problems; headache; irritation of the skin, eyes and mucous membranes; nausea; dizziness and psychological symptoms [4,5]. Moreover, genotoxic damage and endocrine alterations were also detected in a few reports [3–5]. To date, long-term oil effects are scarcely present in literature, only persistent respiratory symptoms, and elevated markers of airway injury in breath condensate, as well as genotoxic and endocrine effects in clean-up workers of the *Prestige* oil spill which have been previously described [14–18].

Most of the genotoxic studies have been done on the effect caused by the *Prestige* oil spill [8–16]. After *Prestige* accident, no genotoxic analyses have been conducted on the three other large oil spills, despite its proximity to the coast: *Tasman Spirit* (Pakistan, 2003), *Hebei Spirit* (Korea, 2007) and *Deepwater Horizon* (Mexico, 2010).

Among the oil genotoxic studies, the most common are performed at moment to exposure or few months later. These studies, using different type of biomarkers, have demonstrated an association between exposure to oil and genotoxic damage [6–13]. However, long-term genotoxic studies are scarce because the complexity of collecting blood samples afterwards. So far, only two studies have been published on the effects of long-term oil exposure from *Prestige*, using different biomarkers, with controversial results [14–16]. In one of them, no evidence of genotoxic damage in exposed individuals was reported seven years after the accident, using comet, micronucleus and T-cell receptor mutation assays [16]. In the other study, carried out by our group, a clear association between the degree of exposure and chromosome damage was found in exposed individuals two years after the *Prestige* oil spill (P-2y study) [14,15]. Long-term follow-up studies in these exposed individuals are relevant because the association between structural chromosome alterations and risk of developing cancer [19–24].

The purpose of the present work was evaluate whether chromosome damage (lesions and structural chromosome alterations) of circulating lymphocytes detected in the P-2y study in exposed individuals persists six years later of the accident (P-6y study). The follow-up genotoxic study was performed in 52 exposed and 23 non-exposed individuals previously studied, using the same experimental protocol. More than 7,700 metaphases from standard lymphocytes culture were analyzed using a sequential *Leishman stain*/*G*-*banding technique*.

## Methods

### Participants in the study

In November 2002, an important number of local fishermen participated in the clean-up of the *Prestige* oil spill. Only fishermen were included in our study in order to minimize other occupational sources of exposure that could act as confounders. A questionnaire survey among 6,780 fishermen one year after exposure was performed. Very strict selection criteria for exposed and non-exposed individuals to oil were applied as detailed in a previous report [17]. In brief, fishermen who collaborated with cleaning-up tasks (>15 days, at least four hours per day) when volatile organic compounds were very intensive were classified as exposed individuals. The non-exposed fishermen were those who did not participate in the clean-up activities for reasons other than health. For first genotoxic study (P-2y) the selection criteria exclude smokers (current smokers and ex-smokers) because the association between smoking and chromosome damage previously described [25,26]. In addition we also excluded individuals with cancer or infertility. A more detailed selection of participants in genotoxic P-2y study was described in Rodriguez-Trigo et al. [14]. A total of 137 individuals were analyzed (91 exposed and 46 non-exposed) [14,15]. The samples for P-2y study were obtained between September 2004 and February 2005, 22 to 27 months after the spill.

**Fig 1:**
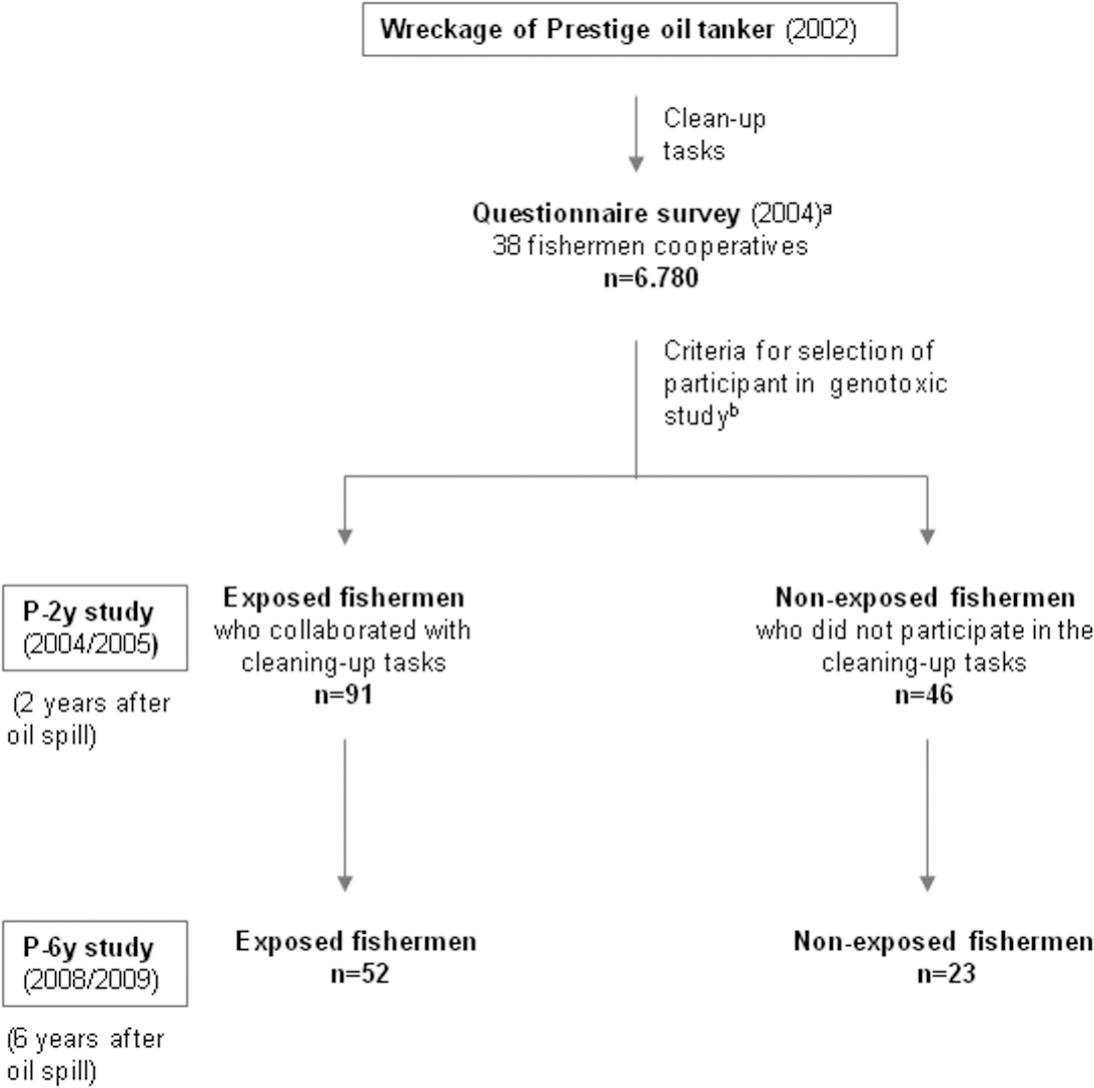
Flow diagram of the study. ^a^Detailed description in Zock et al. 2007. ^b^Detailed description in Rodriguez-Trigo et al. 2010.

In the genotoxic follow-up study (P-6y) only 75 fishermen who had been included in the P-2y study accepted the offer to participate (52 exposed and 23 non-exposed). The exposed group was 15 men and 37 women and the average age was 49.3 years (ranging from 29.2 to 64.4).The non-exposed group was 2 men and 21 women and the average age was 52.2 years (ranging from 31.1 to 63.9). Fig 1 shows the flow diagram of the study. The peripheral blood extraction, transport and procedure of the samples were carried out from November 2008 to April 2009 (from six to six-and-a-half years after the oil spill) following the protocol described previously [14,15].

This Project has been approved by the Ethics Committee of Clinical Research of Galicia, Spain, and all participants signed the participating consent.

The control group used in this work, which was described previously [27], includes ten individuals working in a university lab, 600 km away from the *Prestige* accident. The individuals were six men and four women with ages ranging between 21 and 42 years, and all individuals had never smoked, nor had they been exposed to *Prestige* oil or any other known genotoxic factor.

### Cytogenetic Analysis

Peripheral blood was extracted and circulating lymphocytes cultured for 72 hours in RPMI-1640 medium (GIBCO Invitrogen Cell Culture, Invitrogen, Carlsbad, California) and then harvested according to standard procedures [14,15]. Assessment of chromosome damage included evaluation of chromosome lesions (gaps and breaks) and structural chromosome alterations (such as acentric fragments, deletions, translocations, marker chromosomes). Only high-quality metaphases were studied.

For chromosome lesions analysis, preparations were uniformly stained with Leishman. For each participant, at least 100 metaphases were investigated. The same preparations were stained and banded using Wright staining for detecting structural chromosome alterations. At least 25 G-banded karyotyped for each individual were realized.

All slides were coded before cytogenetic analysis so that information about whether individuals were exposed or non-exposed was not available until the completion of data collection. Aberrant metaphases from each individual were determined by two independent observers.

All chromosome lesions and chromosome structural alterations have been classified according to the International System for Human Cytogenetic Nomenclature [28].

### Statistical Analysis

Differences in cytogenetic results (chromosome lesions and structural chromosome alterations) between the exposed and non-exposed groups adjusted by age were evaluated using a generalized estimating equation, GEE method [29,30]. The GEE approach is an extension of generalized linear models designed to account for repeated, within-individual measurements. This technique is particularly indicated when the normality assumption is not reasonable, as which happens, for instance, with discrete data. The GEE model was used instead of the classic Fisher exact test because the former takes into account the possible within-individual correlation, whereas the latter assumes that all observations are independent. Since several metaphases were analyzed per individual, the GEE model is more appropriate. The GEE model was fitted using the REPEATED statement in the GENMOD procedure. The conservative Type 3 statistics score was used for the analysis of the effects in the model. For comparisons between time periods in exposed and non-exposed individual groups the non-parametric Wilcoxon signed-rank test was used. Statistical significance was set at p<0.05. Statistical analyses were carried out with SAS/STAT release 9.02 (SAS Institute Inc; Cary, NC).

## Results

All individual had a normal constitutional karyotype (46,XX or 46,XY), except one exposed individual with a polymorphic inversion of chromosome 9 in her karyotype, 46,XX,inv(9)(p11q12).

The follow-up cytogenetic study (P-6y) was performed in 52 exposed and 23 non-exposed individuals previously studied (P-2y). The analysis of genotoxic oil effect was performed using two biomarkers: chromosome lesions and chromosome structural alterations.

In present P-6y study, a total of 5,346 and 2,385 metaphases were analyzed from exposed and non-exposed individuals respectively. No significant statistical differences were observed between exposed and non-exposed individuals for chromosome lesions (P = 0.2879) or structural chromosome alterations (P = 0.9263).

Chromosome damage in exposed and non-exposed to oil individuals obtained in the two studies, P-2y and P-6y, is showed in Table 1. In comparing the results obtained in exposed individuals from both studies, chromosome lesions were higher in the P-6y study (P<0.0001) and no differences in structural chromosome alterations were observed (P = 0.1939). For non-exposed, significant increases in lesions and structural chromosome alterations were detected in the P-6y study vs. the P-2y study (P = 0.0289 and 0.0086, respectively). In both studies the unbalanced structural chromosomes were the most frequent alterations in all individuals analyzed.

**Table 1:** Chromosomal lesion and structural chromosome alterations in exposed and non-exposed individuals detected two and six years after oil spill.

		Previous P-2y study			Follow-up P-6y study		
		(2 years after oil exposure)			(6 years after oil exposure)		
		Exposed	Non-exposed	P-value	Exposed	Non-exposed	P-value
**Individuals studied**		**52**	**23**		**52**	**23**	
**Total metaphases analyzed (uniform stain)**		**5392**	**2438**	** **	**5346**	**2385**	** **
**CHROMOSOME LESIONS** (%)		**43 (0.8)**	**26 (1.1)**	**0.3287**	**112 (2.1)**	**70 (2.9)**	**0.3017**
	Chromatid breaks	10	6		4	4	
	Chromosome breaks	13	6		27	12	
	Chromatid gaps	14	10		57	35	
	Chromosome gaps	6	4		24	19	
**Total karyotypes analyzed (G-Banded)**		**1377**	**629**		**1481**	**654**	
**STRUCTURAL CHROMOSOME ALTERATIONS** (%)		**125 (9.1)**	**13 (2.1)**	**0.0007**	**99 (6.7)**	**43 (6.6)**	**0.9493**
**Balanced structural chromosome alterations**		**8**	**4**		**1**	**0**	
	Reciprocal translocations	6	4		1	0	
	Robertsonian translocations	2	0		0	0	
**Unbalanced structural chromosome alterations**		**117**	**9**	**0.0005**	**98**	**43**	**0.9805**
	Deletions	16	4		40	18	
	Deletions and acentric fragments	4	0		5	0	
	Acentric fragments	31	0		6	4	
	Unbalanced translocations	10	1		10	1	
	Dicentric translocations	3	0		6	0	
	Dicentric translocations and deletions and acentric fragments	2	1		0	0	
	Translocations and deletions	0	0		0	3	
	Ring chromosomes	7	0		3	2	
	Marker chromosomes	44	3		28	15	

Statistical differences between exposed and non-exposed individuals are adjusted by age

Cytogenetic results for each individual are shown for exposed and non-exposed individuals in supplementary data

## Discussion

Genotoxic oil exposure studies are limited; most of them carried at the moment of exposure or a few months later and long-term clinical implications are still unknown. The present work is the first genotoxic follow-up study carried out in the same individuals, exposed and non-exposed, four years after of the previous study which itself had been done two years after the *Prestige* accident using chromosome lesions and structural chromosome alterations as biomarkers.

### Chromosome damage in exposed and non-exposed individuals six years after oil exposure

**Fig 2:**
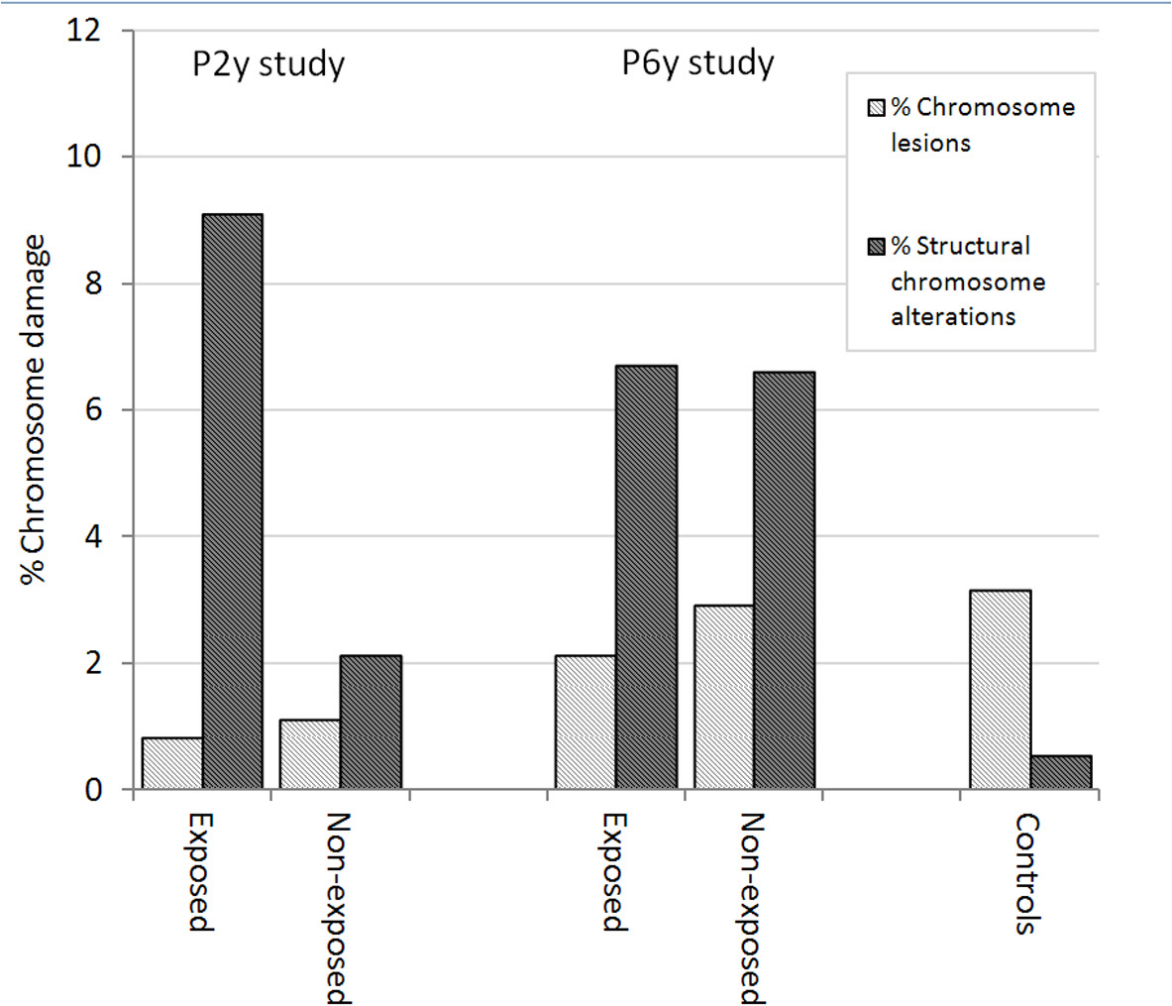
Chromosome damage in exposed and non-exposed individuals from P2y and P6y genotoxic studies and in control individuals.

The frequency of chromosome lesions detected in the P-6y study is similar in exposed and non-exposed individuals, as observed in previous the P-2y study (Fig 2). Up to now, only a few genotoxic studies used chromosome lesions as a biomarker [30–38]. Most of these studies have not detected a relationship between chromosome lesions and genotoxic agent exposure. These findings seem to indicate that chromosome lesions are not a useful biomarker for long-term genotoxic studies (more than two years after exposure). For this reason, the results obtained using this biomarker was not included in the discussion of follow-up study (P6y vs. P2y).

In the P6y study the frequency of the structural chromosome alterations in exposed individuals (6.7%) was similar to that observed in non-exposed individuals (6.6%). These findings suggest that oil toxicity disappears after seven years. In a recent study performed seven years after oil *Prestige* exposure, using biomarkers other than those employed in our study (comet, micronucleus and T-cell receptor mutation assays) [16] no differences have been described between exposed and control individuals. However for two of the three biomarkers (comet and micronucleus assays) used in this work the sensibility to detect long-term genotoxic effects has not been tested. The comet assays detect early biological effects and micronucleus assays have not been performed for long-term genotoxic analyses after occasional exposure [39,40].

The incidence of chromosome structural alterations detected in exposed individuals six years after oil exposure (P6y-study) decreases in relation to the previous study (P2y-study), but no statistical significant differences were found between the two studies (see Fig 2). Moreover, this chromosome damage is at least twice as high as that described in healthy non-smokers controls analyzed by our group (3.15%) [27] or by other groups (0.35%–4.6%) with an average of 2.1% [41,42]. The increase in frequency of chromosome damage in exposed individuals compared to the control individuals indicates the persistence of chromosome damage in exposed individuals six years after the *Prestige* accident (Fig 2). This persistence suggests either: a) some toxic component within the oil could remain inside the body for many years and/or b) the stem cells of the bone marrow had been affected by oil exposure. The second hypothesis is based on this study having been conducted six years after exposure to oil when most of the peripheral blood lymphocytes have been renewed because their half-life is estimated at about four to six years [43]. Accordingly, is has been reported that benzene, one of the main components of oil, is capable of inducing chromosome alterations in stem cells of the bone marrow [44]. The clinical consequences are relevant because the involvement of bone marrow cells increases the risk of leukemia and lymphomas. Moreover, it is know that individuals chronically exposed to benzene have a 20-fold increased risk of developing cancer compared with the general population, particularly hematologic cancer [24]. Our findings suggest that high dose accidental oil exposure may be involved in the origin of cancers caused by chromosome damage (especially by the unbalanced structural chromosomes). However, this suggestion should be considered with caution due to the sample size and the possibility of some kind of selection bias should be considered. Thus, the clinical relevance of accidental oil exposure and its association with increased risk of cancer (assessed by chromosome damage) must be confirmed by further long-term, follow-up genotoxic studies in order to detect cancer early in individuals exposed to oil.

### Increase in chromosome damage in non-exposed individual six years after oil exposure

Unexpectedly, an increase in chromosome damage in non-exposed individuals was detected four years after of initial study (6.6% in P6y vs. 2.1 in P2y). Furthermore, when comparing the chromosomal damage non-exposed and control individuals in both genotoxic studies, a significant increase in the follow-up study (at least two times higher) compared to the level observed in controls was observed, whereas in the previous study the non-exposed and control groups had similar values. This finding is consistent with the increase in respiratory symptoms and markers of oxidative stress observed in these same non-exposed individuals [18].

We do not know the reason for the increase in chromosome damage in non-exposed individuals found in this study, but there is the possibility of indirect exposure to some compounds of oil or other toxic agents during the last four years. One candidate to explain this indirect exposure would be improper storage of oil collected during the clean-up tasks. Once having finished the P6y study we discovered that around 60,000 tons of oil from the *Prestige* had been stored within 50 km of the fishermen’s cooperatives, from which the non-exposed individuals were selected [14,15,17]. Another possible source of indirect oil exposure would be through the ingestion of contaminated food. All exposed and non-exposed individuals in both studies (P-2y and P-6y) were fishermen, so it is likely that their diet is rich in locally-caught fish and seafood. Some of the chemicals from the oil, such as polycyclic aromatic hydrocarbons and heavy metals, are resistant to degradation and accumulate in bottom-dwelling organisms, like mussels and shellfish [45–49]. When these organisms are consumed, these toxic substances accumulate in human tissue which could cause long-term damage. This hypothesis is supported by studies of rats fed mussels contaminated by the oil, which show an increase in genotoxic damage [50,51]. Moreover, the possibility of other additional sources of exposure to toxic agents cannot be excluded. More studies are needed to confirm the presence of chromosome damage in non-exposed fishermen because of the association between increased chromosomal damage and an increased risk of cancer.

For the same reasons as those mentioned above, it is possible that the persistence of chromosome damage observed in exposed individuals could be the result of oil exposure both during the clean-up and from contaminated food ingestion.

## Conclusion

The present work is the first follow-up genotoxic study carried out in circulating lymphocytes to determine genotoxic damage evolution between two and six years after oil exposure in a set group of individuals. Structural chromosome alterations in exposed individuals persist six years after exposure to oil, suggesting that the cells of the bone marrow could be affected. In addition, the unexpected increase in chromosome alterations in non-exposed individuals suggests that they have also been indirectly exposed to some oil compounds. Some of chromosomal damage detected in exposed individuals, may be due to indirect oil exposure or other genotoxic agents, as suggested in the non-exposed ones. More long-term studies are needed to confirm the presence of chromosome damage in exposed and non-exposed fishermen due to the association between increased chromosome damage and an increased risk of cancer in order to detect cancer in early stages.

## Supporting Information

S1 Table Cytogenetic data for each individual. (XLS)

S2 Table Cytogenetic data for each individual. (XLS)
